# Study of an FBG-FP Cascaded Optical Fiber Current Transformer Based on Electrostrictive Material Coupling

**DOI:** 10.3390/s25082492

**Published:** 2025-04-15

**Authors:** Cong Chen, Zhongyuan Li, Qichao Chen, Weichao Zhang

**Affiliations:** 1 China Power Huachuang (Suzhou) Electricity Technology Research Co., Ltd., Suzhou 215123, China; cchen@cpibj.com.cn; 2 Electric Power Research Institute, State Grid Heilongjiang Electric Power Co., Ltd., Harbin 150030, China; li8zhongyuan@126.com; 3 School of Electrical and Electronic Engineering, Harbin University of Science and Technology, Harbin 150080, China

**Keywords:** optical fiber current transformer, electrostrictive material, FBG-FP cascaded structure, high sensitivity

## Abstract

Aiming at the issues of low sensitivity and poor resistance to temperature and vibration interference in traditional optical fiber current transformers, as well as the structural complexity of magnetostrictive material-coupled sensors, this paper integrates a high-sensitivity electrostrictive piezoelectric ceramic sensor with an FBG-FP cascaded fiber-optic sensor. This coupling significantly optimizes the sensor structure. By employing orthogonal intensity demodulation to enhance detection sensitivity, and adopting a multi-cycle waveform-averaging method to calculate the DC output light intensity, temperature calibration and compensation are achieved through the correlation between the DC output light intensity and operating points. Experimental results demonstrate that the designed sensor exhibits a detection bandwidth of 0–7 kHz, fully meeting the requirements for power-frequency current detection. Its current measurement range spans 0.15–42 mA, with a minimum measurable current as low as 150 μA. This study provides a compact, high-precision, highly scalable, and adaptable current detection solution for power systems, demonstrating significant engineering application value.

## 1. Introduction

With the rapid development of critical power equipment in current power systems, including total generator installed capacity, long-distance transmission capabilities, and grid scale, the safe and stable operation of power systems faces significant challenges due to the integration of high-penetration renewable energy and power electronic devices. Traditional electromagnetic current transformers fail to meet modern grid requirements for current detection due to their low measurement accuracy, poor smart interconnection capabilities, and reliance on manual inspection methods [1,2,3,4]. Recent advancements in fiber-optic sensing and demodulation technologies have drawn widespread attention to optical current transformers, which offer unique advantages such as high precision, strong immunity to electromagnetic interference, long transmission distances, and rapid parasitic interconnection networking [5,6,7,8].

Traditional fiber-optic current transformers primarily include all-fiber, hybrid electro-optic, and magnetostrictive material-coupled types. Among these, all-fiber current sensors operate based on the Faraday magneto-optic effect. When a current-carrying conductor generates a magnetic field, this field interacts with linearly polarized light in the sensing fiber, causing a rotation of the polarization plane. The rotation angle is proportional to the current intensity, enabling accurate current measurement through detection of this angular shift [9]. Based on optical configurations, all-fiber current sensors are categorized into polarimetric and interferometric structures. The latter further divides into ring-type Sagnac interferometers and reflective Sagnac interferometers. Polarimetric structures convert polarization rotation angles into light intensity signals, which are then processed to derive current values. While these systems benefit from minimal optical components and simple setups—making them suitable for small-scale power systems—they suffer from poor stability, low noise immunity, and significant measurement errors [10,11]. To address the aforementioned issues, in 2000, Briffod F. et al. proposed a configuration incorporating a linear 22.5° Faraday rotator, which enhanced the sensor’s measurement accuracy [12]. In 2017, Zhang H. et al. introduced a sensor combining a single-polarization single-mode (SPSM) coupler with a loop structure. The SPSM coupler simplified the system and improved stability, while the loop structure enhanced sensitivity [13]. In 2019, Li Y.S. et al. eliminated the impact of temperature drift on measurement accuracy by integrating magneto-optic glass to calibrate the fiber output signals, thereby improving both steady-state and transient current measurement precision [14]. The ring-type Sagnac interferometer, first proposed by Nicati et al. in 1988 [15], operates as follows: light from the source is converted into linearly polarized light via a polarizer, then split by a 50:50 coupler into two beams propagating through a fiber coil wound around the current-carrying conductor. Under the magnetic field, both beams acquire current-dependent phase shifts and recombine at the coupler to generate interference patterns. However, this structure suffers from poor stability, weak noise immunity, and vibration sensitivity, making it unsuitable for outdoor power systems. In contrast, the reflective Sagnac interferometer, pioneered by Blake et al. in 1995 [16], evolved from the ring-type design. Compared to earlier interferometric configurations, it offers stronger noise immunity, a larger dynamic range, fewer optical components, and reduced sensitivity to temperature and vibrations, enabling widespread adoption in power systems. In 2004, Takahashi et al. developed a Sagnac sensor using a single-mode fiber pigtail, where a depolarizer was integrated into the Sagnac coil to suppress phase errors and improve accuracy. Experimental results validated its suitability for substation current detection [17]. In 2021, Wu et al. applied a reflective all-fiber current sensor for ship leakage current measurement, achieving high-precision detection in the 1–99 mA range [18]. In summary, while all-fiber current sensors have seen extensive research and application, critical challenges persist: low measurement sensitivity (due to the fiber’s inherent insensitivity to current), accuracy degradation from temperature/vibration effects, long-term stability issues, and bulky configurations hindering installation. These limitations restrict their applicability in most engineering scenarios.

Current sensors based on the magnetostrictive effect typically combine an FBG with Giant Magnetostrictive Material (GMM) to achieve current sensing. When the measured current generates a magnetic field, it drives the GMM to undergo magnetostrictive deformation, which induces a central wavelength shift in the FBG. By demodulating this wavelength shift, the magnitude of the current can be indirectly determined. Due to the structural simplicity and ease of implementation of GMM-FBG coupled current transformers, researchers have focused on enhancing their sensitivity. In 2019, Shuchao Wang proposed a method to apply pre-stress to the GMM, improving its hysteresis characteristics and significantly increasing the sensitivity of GMM-FBG sensors [19]. The same year, Lopez J.D. et al. developed a sensor using a magnetostrictive polymer composite with aligned magnetic domains. By embedding the sensing FBG into a composite block fabricated with only 0.42 g Terfenol-D powder and epoxy resin, their sensor achieved the same measurement range and accuracy as conventional Terfenol-D block-based sensors while incorporating temperature compensation functionality [20]. Jiahong Zhang enhanced the sensor’s detection sensitivity and temperature stability by employing a dual-ring lever mechanism. However, the structural complexity of this design hindered its applicability in practical engineering implementations [21]. Fei Jiao established the correlation between sensitivity enhancement and key parameters such as grating length, refractive index modulation depth, and apodization function, providing comprehensive theoretical analysis and practical guidance for GMM-FBG coupled current transformer design [22]. Concurrently, the current–temperature cross-sensitivity in GMM-FBG coupled systems has been a major research focus. In 2000, Mora et al. achieved simultaneous current and temperature measurement by bonding an auxiliary FBG to a Monel400 alloy (matched to GMM’s thermal expansion coefficient) [23]. In 2003, Chiang et al. realized automatic temperature compensation by adhering a single FBG to both GMM and Monel400 substrates [24]. In 2006, Reilly et al. implemented temperature-independent AC current measurement via feedback-controlled static operating point stabilization [25]. In 2013, Zhao et al. resolved cross-sensitivity using a dual-magnetic-circuit system, exploiting opposite strain responses from two FBGs within the dual magnetic circuits [26]. However, the asymmetric magnetostriction curve of GMMs necessitates the application of an externally applied bias magnetic field to address their linear vibration under alternating magnetic fields and improve magnetic field sensing capability. This renders GMM-based fiber-optic current transformers structurally complex. Additionally, the inherent low microstrain sensitivity of FBGs—an intrinsic limitation in their sensing performance—leads to a critical issue where such fiber-optic current transformers exhibit poor detection capability for weak current signals.

Therefore, this paper addresses the critical issues of low detection sensitivity, poor temperature, and vibration immunity in all-fiber current transformers, as well as the structural complexity of GMM-based coupled fiber-optic current transformers. A fiber-optic current transformer coupling optical fiber with electrostrictive materials is designed, significantly simplifying the sensor structure. By utilizing FBG-FP cascaded sensing technology, the electrostrictive material is coupled with the Fabry–Perot (FP) cavity of the FBG-FP sensor, substantially enhancing detection sensitivity. Additionally, the FBG-FP orthogonal intensity demodulation system is employed to correlate the DC output signal intensity with temperature drift characteristics. Through averaging the processing of the sensor’s AC output signals to obtain the DC intensity, and implementing Proportional Integral Derivative (PID) closed-loop control for temperature compensation, the sensor’s detection stability and anti-interference capability are further improved.

## 2. Research on the Detection Principles of Sensors

### 2.1. FBG-FP Cascaded Spectral Characteristics Analysis

**Figure 1:**
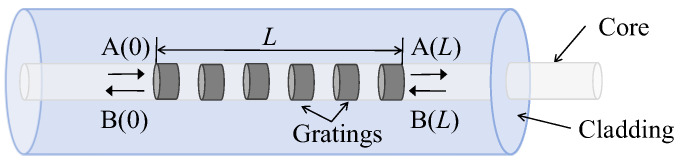
Schematic diagram of FBG structure.

FBG is the abbreviation of fiber Bragg grating. It is formed by irradiating the fiber core with ultraviolet beams to induce periodic variations in the refractive index of the core. Due to the influence of the refractive index, when light waves propagate through the grating, those satisfying the Bragg condition will be selectively reflected while other light waves remain unaffected. Therefore, FBG can achieve optical wavelength selection functionality. The structure of FBG is shown in Figure 1.

The transmission characteristics of FBG can be described using mode-coupling theory, transfer matrix theory, and Fourier transform theory. The first two can accurately analyze the coupling phenomena of light wave propagation in fiber gratings, while the Fourier transform method is only suitable for analyzing FBGs with low reflectivity. However, the mode-coupling equations are relatively complex. To facilitate calculations, the simplified mode-coupling equations can be solved using the transfer matrix. The forward and backward propagating light waves in FBG can be represented in matrix form as follows [27]:

(1) $$\left(\begin{array}{l} A(0)\\ B(0) \end{array}\right)=\left(\begin{array}{cc} S_{11} & S_{12}\\ S_{21} & S_{22} \end{array}\right)\left(\begin{array}{l} A(L)\\ B(L) \end{array}\right)$$

The transmission characteristics of FBG can be analyzed using mode-coupling theory to determine the values of each element in the matrix. By applying the boundary conditions A(0) = 1 and B(L) = 0, the reflection and transmission coefficients of the FBG can be solved. The values of the matrix elements are as follows:

(2) $$S_{11}=\cosh\left(SL\right)-i\left(\frac{\Delta \beta }{S}\right)\sinh\left(SL\right)$$

(3) $$S_{12}=-i\left(\frac{k}{S}\right)\sinh\left(SL\right)$$

(4) $$S_{21}=i\left(\frac{k}{S}\right)\sinh\left(SL\right)$$

(5) $$S_{22}=\cosh\left(SL\right)+i\left(\frac{\Delta \beta }{S}\right)\sinh\left(SL\right)$$

where *S* = [*k*^2^ − (Δ*β*)^2^]^1/2^, the coupling coefficient of the grating *k = π*∆*n*/*λ*_B_, Δ*β* = 2*nπ*/*λ* − 2*nπ*/*λ*_B_, Bragg wavelength *λ*_B_ = 2*n*_eff_Λ, with *n*_eff_ and Λ being the effective refractive index and period of the grating, *L* the grating length, Δ*n* the refractive index modulation depth, and *n* the fiber refractive rate.

The reflection coefficient is *r_g_* = *S*_21_/*S*_11_ = |*r_g_*|*exp*(*iφ_r_*), and the transmission coefficient is *t*_g_ = 1/*S*_11_ = |*t*_g_|exp(*iφ*_t_). The reflection coefficient, transmission coefficient, and their corresponding phase angles can be determined as follows:

(6) $$\left|r_{g}\right|=\frac{k\sinh\left(SL\right)}{{\left[\Delta \beta^{2}\sinh^{2}\left(SL\right)+S^{2}\cosh^{2}\left(SL\right)\right]}^{1/2}}$$

(7) $$\phi_{r}=\pi +\arctan\frac{S\cosh\left(SL\right)}{\Delta \beta \sinh\left(SL\right)}$$

(8) $$\left|t_{g}\right|=\frac{S^{2}}{{\left[\Delta \beta^{2}\sinh^{2}\left(SL\right)+S^{2}\cosh^{2}\left(SL\right)\right]}^{1/2}}$$

(9) $$\phi_{t}=-\frac{\pi }{2}-\beta_{0}L+\arctan\frac{S\cosh\left(SL\right)}{\Delta \beta \sinh\left(SL\right)}$$

The reflectivity and transmissivity of the FBG are expressed as

(10) $$R_{g}=\frac{k^{2}\sinh^{2}(SL)}{\Delta \beta^{2}\sinh^{2}(SL)+S^{2}\cosh^{2}(SL)}$$

(11) $$T_{g}=\frac{S^{2}}{\Delta \beta^{2}\sinh^{2}(SL)+S^{2}\cosh^{2}(SL)}$$

**Figure 2:**
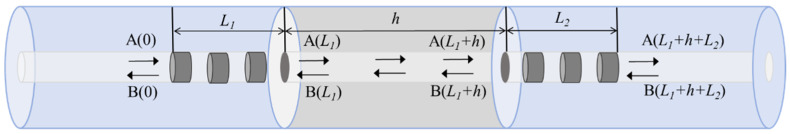
Schematic diagram of FBG-FP cavity structure.

The FBG-FP cavity is formed by inscribing two gratings with identical central wavelengths on the same optical fiber. The two gratings act as reflectors for the FP interferometer, with grating lengths *L*_1_ and *L*_2_, and an FP cavity length *h*. The structure of the FBG-FP cavity is shown in Figure 2.

Substituting the grating reflection coefficient r^*^ = *S*_21_/*S*_11_ and transmission coefficient t* = 1/*S*_11_ into Equation (1) yields

(12) $$\left(\begin{array}{c} A(0)\\ B(0) \end{array}\right)=\left(\begin{array}{cc} 1/t & r^{*}/t^{*}\\ r/t & 1/t^{*} \end{array}\right)\left(\begin{array}{c} A(L)\\ B(L) \end{array}\right)$$

Assuming that light waves in standard single-mode fiber undergo only phase changes, then

(13) $$\left(\begin{array}{c} A(L_{1}+h)\\ B(L_{1}+h) \end{array}\right)=\left(\begin{array}{cc} P & 0\\ 0 & P^{-1} \end{array}\right)\left(\begin{array}{c} A(L_{1})\\ B(L_{1}) \end{array}\right)$$

where *P* = *exp*(*iβh*), *β* = *2nπ*/*λ* is the propagation constant of the light wave in the fiber, and *h* is the fiber length.

Thus, the matrix transmission equation for the FBG-FP can be written as

(14) $$\left(\begin{array}{c} A(0)\\ B(0) \end{array}\right)=\left(\begin{array}{cc} 1/t_{1} & r_{1}^{*}/t_{1}^{*}\\ r_{1}/t_{1} & 1/t_{1}^{*} \end{array}\right)\left(\begin{array}{cc} P & 0\\ 0 & P^{-1} \end{array}\right)\left(\begin{array}{cc} 1/t_{2} & r_{2}^{*}/t_{2}^{*}\\ r_{2}/t_{2} & 1/t_{2}^{*} \end{array}\right)\left(\begin{array}{c} A(L_{1}+h+L_{2})\\ B(L_{1}+h+L_{2}) \end{array}\right)$$

The transfer matrix can be expressed as

(15) $$T=\left(\begin{array}{cc} T_{11} & T_{12}\\ T_{21} & T_{22} \end{array}\right)=\left(\begin{array}{cc} P/(t_{1}t_{2})+r_{1}^{*}r_{2}/(Pt_{1}^{*}t_{2}) & Pr_{2}^{*}/(t_{1}t_{2}^{*})+r_{1}^{*}/(Pt_{1}^{*}t_{2}^{*})\\ Pr_{1}/(t_{1}t_{2})+r2/(Pt_{1}^{*}t_{2}) & P*r_{1}r_{2}^{*}/(t_{1}t_{2}^{*})+1/(Pt_{1}^{*}t_{2}^{*}) \end{array}\right)$$

From the above equation, the reflection and transmission coefficients of the FBG-FP can be derived as

(16) $$r_{F-P}=\frac{T_{21}}{T_{11}}=\left|r_{F-P}\right|\exp\left(i\phi_{r}\right)$$

(17) $$t_{F-P}=\frac{1}{T_{11}}=\left|t_{F-P}\right|\exp\left(i\phi_{t}\right)$$

Assuming the parameters of the two gratings are identical, i.e., |*r*_1_| = |*r*_2_|, |*t*_1_| = |*t*_2_|, *ϕ*_*r*1_ = *ϕ*_*r*2_, the reflection and transmission coefficients can be simplified as

(18) $$\left|r_{F-P}\right|=\sqrt{\frac{2{\left|r\right|}^{2}-2{\left|r\right|}^{2}\cos(2\beta h-2\phi_{t})}{1+{\left|r\right|}^{4}-2{\left|r\right|}^{2}\cos(2\beta h-2\phi_{t})}}$$

(19) $$\left|t_{F-P}\right|=\sqrt{\frac{{\left|t\right|}^{4}}{1+{\left|r\right|}^{4}-2{\left|r\right|}^{2}\cos(2\beta h-2\phi_{t})}}$$

Thus, the reflectivity and transmissivity are

(20) $$R_{F-P}={\left|r_{F-P}\right|}^{2}=\frac{2{\left|r\right|}^{2}-2{\left|r\right|}^{2}\cos(2\beta h-2\phi_{t})}{1+{\left|r\right|}^{4}-2{\left|r\right|}^{2}\cos(2\beta h-2\phi_{t})}$$

(21) $$T_{F-P}={\left|t_{F-P}\right|}^{2}=\frac{{\left|t\right|}^{2}}{1+{\left|r\right|}^{4}-2{\left|r\right|}^{2}\cos(2\beta h-2\phi_{t})}$$

Let the finesse be denoted as $F=\frac{4{\left|r\right|}^{2}}{{\left[1-{\left|r\right|}^{2}\right]}^{2}}$, then the above equation can be rewritten as

(22) $$R_{F-P}=\frac{F\sin^{2}(\beta h-\phi_{r})}{1+F\sin^{2}(\beta h-\phi_{r})}$$

(23) $$T_{F-P}=\frac{1}{1+F\sin^{2}(\beta h-\phi_{r})}$$

**Figure 3:**
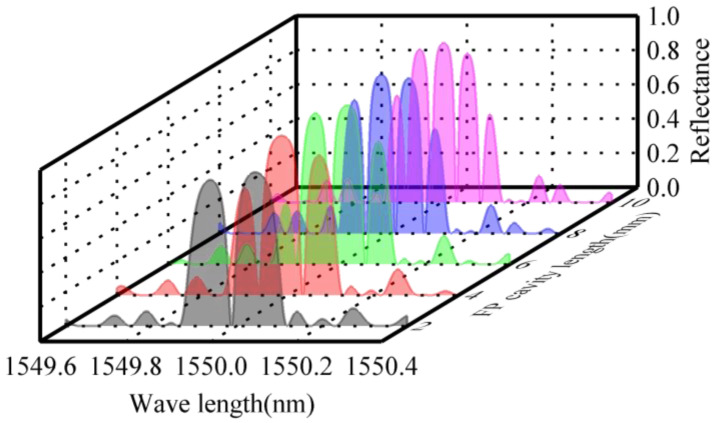
Reflection spectra of FBG-FP with different FP cavity lengths.

Since the grating length and FP cavity length influence the reflection spectrum characteristics of the FBG-FP cavity, thereby affecting the sensor’s sensitivity and demodulation accuracy, calculations are performed to analyze the impact of these parameters on the reflection spectrum. With the effective refractive index *n_eff_* = 1.456, grating period *Λ* = 532.28 nm, refractive index modulation depth Δ*n* = 1 × 10^−4^, and grating length *L* = 5 mm, the reflection spectra of the FBG-FP cavity under different FP cavity lengths are shown in Figure 3. The results indicate that as the FP cavity length increases, the reflection bandwidth and maximum reflectivity of the FBG remain unchanged. However, the number of resonance peaks within the FBG reflection bandwidth increases, and the spacing between resonance peaks decreases.

### 2.2. Analysis of Coupling Sensing Characteristics Between Stacked Piezoelectric Ceramics and FBG-FP

The comprehensive performance parameters of PZT sensors directly affect the detection performance of fiber-optic current transformers. Common performance parameters of PZT are listed in Table 1. The PZT series primarily include PZT-4, PZT-5, and PZT-8, each exhibiting distinct mechanical quality factors and piezoelectric strain constants, corresponding to different application scenarios. PZT-4 and PZT-8 feature high mechanical quality factors but low piezoelectric strain constants, making them suitable for applications with high-voltage or high-strain input excitation. Conversely, PZT-5 has a low mechanical quality factor but a high piezoelectric strain constant, enabling larger deformation displacement under the same driving voltage. This characteristic makes it highly suitable for applications utilizing the inverse piezoelectric effect.

Lead zirconate titanate (PZT) is a typical ferroelectric material containing numerous electric domains. In its unpolarized state, these domains are randomly distributed, resulting in no overall piezoelectric effect. After high-voltage polarization, the electric domains realign along the electric field direction under strong electric fields, forming a stable polarization direction. When the external electric field aligns with the polarization direction, the material elongates; conversely, it contracts, exhibiting mechanical deformation consistent with the electric field direction.

Bulk-structured PZT with significant thickness exhibits excellent mechanical strength and can withstand high stress, but requires higher driving voltages to generate sufficient electric field intensity. In contrast, thin-plate PZT, despite lower mechanical strength, can produce larger electric fields under lower voltages, enabling significant mechanical deformation at low operating voltages. Considering that PZT-based fiber-optic current transformers do not require external force bearing or power output, this study adopts polarized thin-plate PZT stacked with electrode layers. The stacked piezoelectric ceramic sensor is fabricated using a low-voltage stacked co-firing process, yielding a structure with exceptional electrostrictive performance and service life, ideal for long-term power-frequency current detection in fiber-optic current transformers.

**Figure 4:**
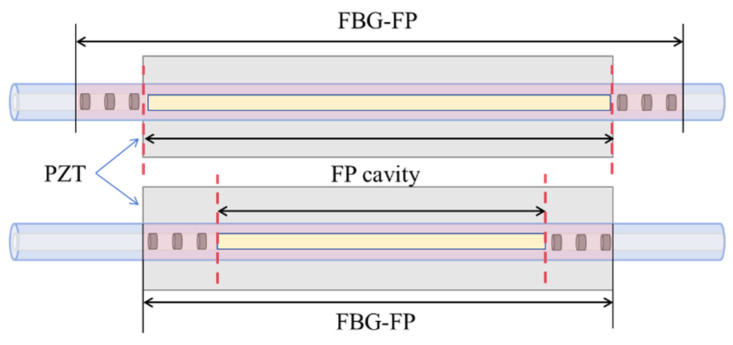
Two bonding configurations of FBG-FP.

The FBG-FP transduction unit is formed by bonding the FBG-FP to the PZT surface using epoxy adhesive. Two bonding configurations exist: one involves bonding only the central FP cavity to the PZT surface, while the other bonds both FBGs and the FP cavity to the PZT surface. The two bonding configurations of the FBG-FP are illustrated in Figure 4.

The distinction between these two configurations lies in whether the extensional strain from the PZT acts solely on the FP cavity of the FBG-FP sensor or simultaneously on both the FP cavity and the two FBGs at the ends. For the bonding configuration where only the FP cavity is attached, the strain generated by the PZT acts exclusively on the FP cavity. This strain alters both the effective refractive index of the FP cavity’s fiber core and the FP cavity length, as described by [28]:

(24) $$l_{FP}^{\prime }=l_{FP}(1+\varepsilon )$$

(25) $$n_{\mathrm{eff}}^{\prime }=n_{\mathrm{eff}}(1-\varepsilon P_{e})$$

where *l’_FP_* and *l_FP_* are the FP cavity lengths before and after the application of PZT-induced strain, respectively; ε is the strain generated by the PZT; *n′_eff_* and *n_eff_* are the effective refractive indices of the FP cavity’s fiber core before and after strain application, respectively; and *P_e_* is the effective elasto-optic coefficient of the fiber.

Substituting Equations (24) and (25) into Equation (23) yields the reflectivity expression of the FBG-FP under strain:

(26) $$R_{FBG-FP}=\frac{2R_{g}[1-\cos\frac{4\pi n_{\mathrm{eff}}^{\prime }l_{FP}^{\prime }}{\lambda }]}{1+{R_{g}}^{2}-2R_{g}[1-\cos\frac{4\pi n_{\mathrm{eff}}^{\prime }l_{FP}^{\prime }}{\lambda }]}$$

**Figure 5:**
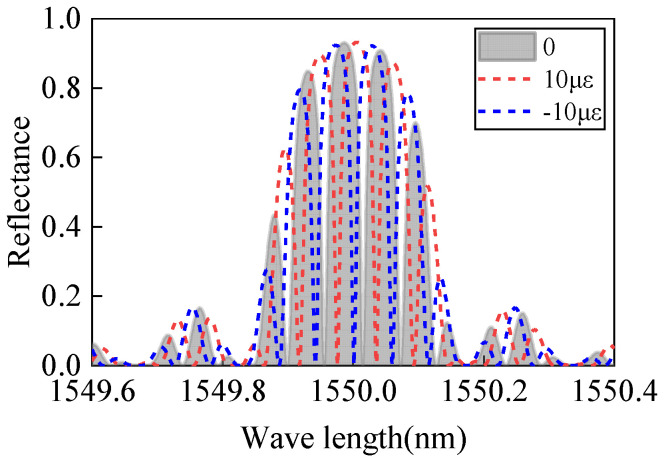
Spectrum of FBG-FP under negative strain.

Using Equation (26), the strain-affected FBG-FP spectrum is calculated with the following parameters: FBG central wavelength *λ* = 1550 nm, refractive index modulation dept Δ*n* = 1 × 10^−4^, grating length is 5 mm, FP cavity length is 10 mm, and the applied strain is ±10με (positive strain for PZT extension, negative strain for PZT compression). The calculated results are shown in Figure 5.

For the bonding configuration where both FBGs and the central FP cavity are attached to the PZT surface, the strain generated by the PZT affects both the FBGs and the FP cavity. In this case, the strain not only alters the effective refractive index *n_eff_* of the FP cavity’s fiber core and the FP cavity length *l_FP_*, but also modifies the effective refractive index *n_eff1_* and grating period *Λ* of the FBGs, given specifically below:

(27) $$l_{FP}^{\prime }=l_{FP}(1+\varepsilon )$$

(28) $$n_{\mathrm{eff}}^{\prime }=n_{\mathrm{eff}}(1-\varepsilon P_{e})$$

(29) $$\Lambda^{\prime }=\Lambda (1+\varepsilon )$$

(30) $$n_{\mathrm{eff}1}^{\prime }=n_{\mathrm{eff}1}(1-\varepsilon P_{e})$$

**Figure 6:**
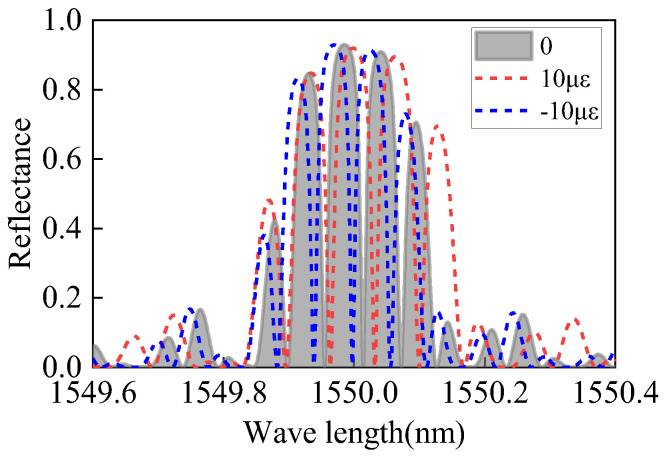
Spectrum of FBG-FP under positive strain.

Compared to the first bonding configuration, under strain, the effective refractive index and grating period of the FBGs change, leading to a shift in the central wavelength of the FBGs and corresponding variations in their reflectivity. Consequently, the central wavelength of the FBG-FP within the FBG envelope also shifts, and its reflectivity adjusts according to the FBG changes. Using the above equations, the interference spectrum of the FBG-FP sensor under the same strain is calculated, and the results are shown in Figure 6.

Comparing the spectral changes under positive strain for both bonding configurations reveals that when only the FP cavity is strained, the maximum reflectivity remains near 93.1%. However, when both the FP cavity and FBGs are strained, the maximum reflectivity decreases (e.g., to 91.9% at 10 *με*). The configuration bonding only the FP cavity exhibits minimal impact on the linear operating range and sensitivity of the resonance peaks. Additionally, this configuration requires a smaller PZT volume while maintaining the FBG-FP dimensions, facilitating sensor miniaturization. Therefore, this study selects the bonding configuration where only the FP cavity is attached to the PZT surface.

### 2.3. Optimization Design of Electromagnetically Coupled Stacked Piezoelectric Ceramic Driving Structure

**Figure 7:**
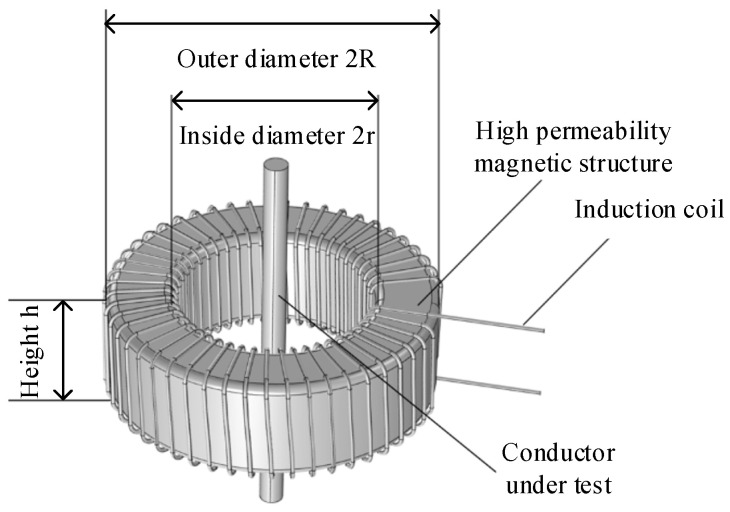
Diagram of magnetic coupling structure.

The piezoelectric ceramic sensor acquires its driving voltage from a magnetic coupling structure sleeved around the measured conductor. The magnetic coupling structure consists of a magnetic core and an induction coil, as shown in Figure 7. The magnetic field generated by the measured current induces a voltage in the induction coil through the coupling effect of the magnetic core, thereby driving the PZT to produce strain. Consequently, the magnetic coupling structure is a critical component determining whether the FBG-FP current sensing system can achieve small-current measurements, significantly influencing the system’s minimum measurable current and measurement range. To enable the FBG-FP current sensing system to measure small currents, the magnetic coupling structure must generate a sufficiently large induced voltage even under low-current conditions. The optimal magnetic coupling structure is determined by analyzing the magnetic core and induction coil separately.

**Figure 8:**
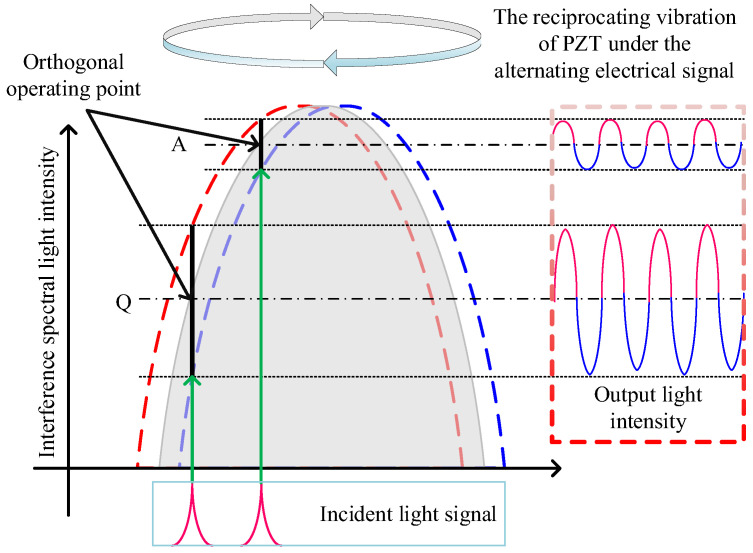
Schematic diagram of intensity demodulation.

**Figure 9:**
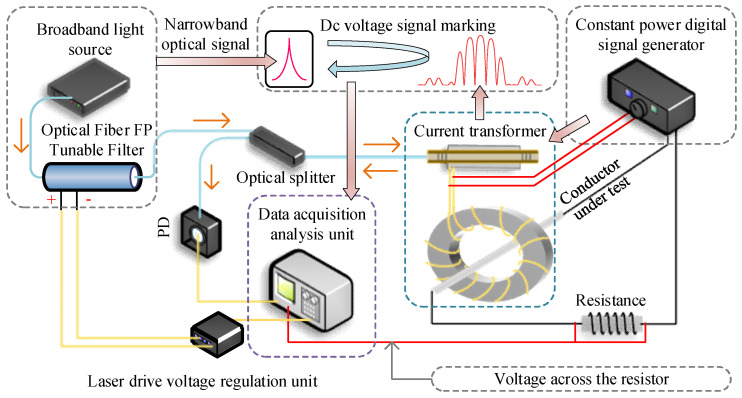
Schematic diagram of FBG-FP sensor sensing detection and demodulation system.

**Figure 10:**
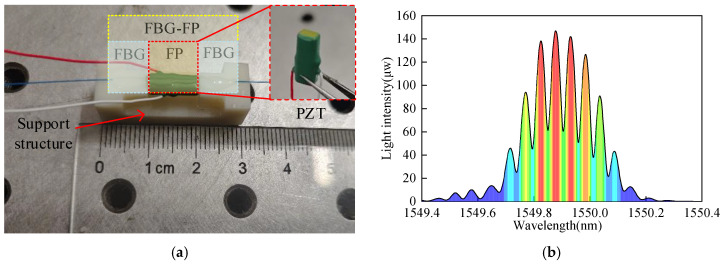
Sensor and its physical diagram: (**a**) a physical diagram of the sensor, (**b**) the interference spectrum of the sensor.

**Figure 11:**
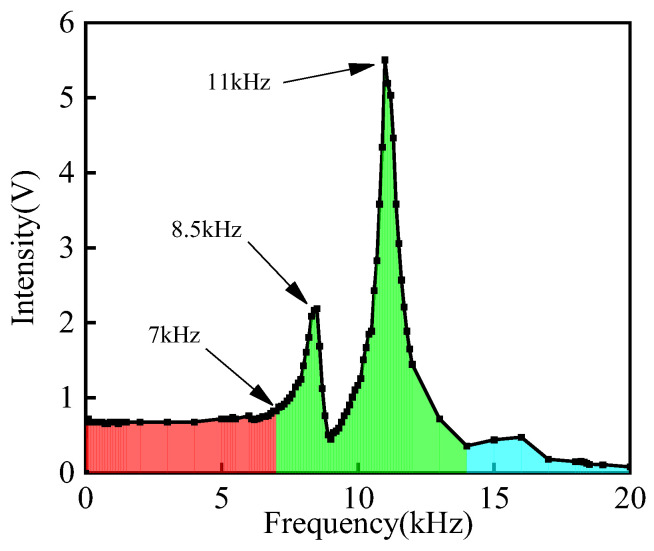
Amplitude–frequency characteristic curve.

**Figure 12:**
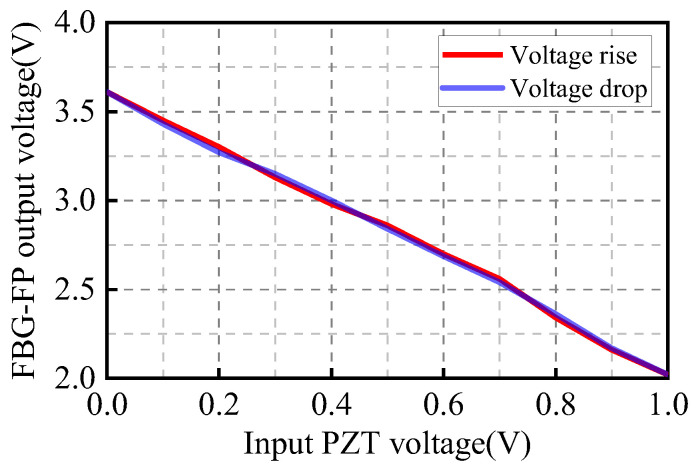
Experimental results of PZT.

For the magnetic coupling structure shown in Figure 3, Figure 4, Figure 5, Figure 6, Figure 7, Figure 8, Figure 9, Figure 10, Figure 11 and Figure 12, let its inner diameter be 2*r_in_*, outer diameter 2*R_out_*, height *h_m_*, permeability *μ*_0_, and number of induction coil turns *N_m_*. When an alternating current *I_in_* flows through the central conductor, the magnetic flux density at the inner diameter of the magnetic core is

(31) $$B\left(r_{\mathrm{in}}\right)=\frac{\mu_{0}I_{\mathrm{in}}\sin\left(\omega t\right)}{2\pi r_{\mathrm{in}}}$$

Integrating the above equation over the cross-sectional area yields the magnetic flux as

(32) $$\phi =\int_{S}B(r_{\mathrm{in}})dr=\int_{r_{\mathrm{in}}}^{R_{\mathrm{out}}}\frac{\mu_{0}h_{m}I_{\mathrm{in}}\sin\omega t}{2\pi r_{\mathrm{in}}}dr=\frac{\mu_{0}I_{\mathrm{in}}h_{m}}{2\pi }\ln(\frac{R_{\mathrm{out}}}{r_{\mathrm{in}}})\sin\omega t$$

The induced voltage in the coil is calculated using Faraday’s law of electromagnetic induction:

(33) $$E=-\frac{d\phi }{dt}=-\frac{N\mu_{0}I_{\mathrm{in}}h_{m}\omega }{2\pi }\ln(\frac{R_{\mathrm{out}}}{r_{\mathrm{in}}})\cos\omega t$$

From the above equations, it can be observed that the dimensions of the magnetic core exhibit minimal influence on the induced voltage generated by the coil. The induced voltage output primarily depends on the number of coil turns *N* and the permeability *μ*_0_ of the magnetic core. For a constant input current in the central conductor, increasing the number of coil turns or selecting magnetic core materials with higher permeability can effectively enhance the induced voltage output. This drives the PZT to generate larger strain, which is advantageous for the FBG-FP sensing system to achieve small-current measurements.

To enable the coil to generate relatively higher induced voltage and improve measurement accuracy, this study employs a toroidal magnetic core fabricated from ultra-microcrystalline alloy with higher permeability and saturation magnetic flux density. The core is insulated using cable paper tape, with geometric dimensions as follows: inner diameter, 5.1 cm; outer diameter, 8.3 cm; and height, 2.6 cm. Based on the actual core dimensions, the coil parameters are estimated: the inner circumference of the magnetic core C = 2πr = 160.22 mm, and the outer diameter of the enameled wire used for winding is 0.72 mm. Under single-layer winding constraints, the maximum number of turns is calculated as 222. To enhance the sensor’s capability for detecting small currents, the maximum number of turns is adopted for winding.

## 3. Sensor Fabrication and Experimental Study

### 3.1. Temperature Calibration Method for Sensors Based on Quadrature Intensity Demodulation

Fiber-optic sensors commonly employ two demodulation approaches: phase demodulation and quadrature intensity demodulation. Phase demodulation is more suitable for fiber-optic sensors utilizing two-beam interferometry, such as Mach–Zehnder or Sagnac interferometers. In contrast, quadrature intensity demodulation is better suited for multi-beam interferometric fiber-optic sensors with FP cavities. Therefore, this study adopts quadrature intensity demodulation for signal detection in the FBG-FP cascaded fiber-optic current transformer. This demodulation method employs a narrowband optical signal as the light source, incident on the spectral sideband of the sensor. The strain signal is demodulated within the linear region of the spectral sideband, as illustrated in Figure 8. Under sinusoidal strain excitation, the spectrum oscillates sinusoidally around the operating point, producing a sinusoidal electrical signal after demodulation. When no strain is applied, the demodulated output is a DC signal. Regarding the selection of the static operating point, it is typically set at the maximum slope location of the interference spectrum curve, where the highest sensor sensitivity and maximized linear dynamic range are achieved. If the static operating point is selected at point A, deviating from the maximum slope location Q, it not only significantly reduces sensitivity but also introduces nonlinear errors into the detection system. This phenomenon arises from the inherent nonlinear variation in the slope of the FP sensor’s interference spectrum within its longitudinal mode spacing interval, which is an intrinsic characteristic of FP sensor interference spectra.

When ambient temperature changes, the cavity length of the sensor’s Fabry–Pérot (FP) cavity is altered, causing a drift in the optimal static operating point of the sensor’s interference spectrum. This results in a mismatch between the optimal static operating point and the central operating wavelength of the narrowband optical signal, leading to unstable detection performance of the sensor. Therefore, it is necessary to adjust the central operating wavelength of the narrowband optical signal to ensure continuous alignment with the optimal static operating point of the sensor’s interference spectrum. To meet the requirement for adjusting the central operating wavelength of the incident driving optical signal, this study employs a combination of a broadband optical signal and a fiber-optic FP tunable filter to generate a narrowband optical signal. The principle involves filtering the broadband optical signal through the fiber-optic FP tunable filter to emit a narrowband optical signal. By applying a voltage to the fiber-optic FP tunable filter, the displacement of the internal piezoelectric ceramic actuator controlling the FP cavity length is adjusted, thereby regulating the central operating wavelength of the transmitted narrowband optical signal. This study utilizes PID closed-loop feedback control to dynamically adjust the fiber-optic FP tunable filter, enabling the central operating wavelength of the narrowband optical signal incident on the sensor to track the sensor’s optimal static operating point Q. The principle is as follows: the narrowband optical signal, after reflection from the sensor, is converted into a DC voltage signal by the photodetector. Assuming the central operating wavelength of the narrowband optical signal aligns with the sensor’s optimal static operating point Q, the DC voltage signal remains unchanged if no external action occurs. Thus, the amplitude of the DC voltage signal corresponds to the narrowband optical signal. When the sensor detects an AC current signal, the output voltage signal from the photodetector becomes an AC voltage signal, which still retains a DC voltage component. Specifically, the sum of the maximum and minimum values of the AC voltage signal equals twice the DC component. According to the intensity demodulation principle, when environmental temperature shifts the optimal static operating point Q, the DC component of the sensor’s output signal via the photodetector also changes. At this point, the fiber-optic FP tunable filter is adjusted to alter the central operating wavelength of the emitted narrowband optical signal, thereby realigning the DC component of the sensor’s output signal to match the amplitude corresponding to the optimal static operating point Q. To accurately determine the DC component, the average value of the sensor’s AC output signal over every 10 cycles is calculated, enabling precise adjustment of the fiber-optic FP tunable filter. This achieves temperature compensation for the sensor and completes the system’s closed-loop regulation. The schematic of the sensing and testing system is shown in Figure 9. The broadband optical source used in this study is the ASE-C LIGHT SOURCE, with an output optical signal wavelength range of 1520–1570 nm and an optical power of 100 mW. The fiber-optic FP tunable filter is the FFP-TF2 Tunable Filter provided by MICRON OPTICS (Atlanta, GA, USA). The photodetector is the PDA10CS-EC from THORLABS (Newton, NJ, USA), and the digital signal generator is the UTG932 Standard Function Voltage Signal Generator from UNI-T (Dongwan City, China).

### 3.2. Sensor Fabrication and Detection Bandwidth Testing Study

The fabrication of the sensing probe involves the preparation of the FBG-FP and its encapsulation with PZT. The piezoelectric ceramic material used in this study is lead zirconate titanate (PZT) provided by Core Tomorrow Technology Co., Ltd. (Harbin, China), with a mechanical quality factor of 70, piezoelectric constants *d*_31_ = −290, *d*_33_ = 635, Curie temperature of 150 °C, density of 8000 kg/m^3^, and overall performance comparable to PZT-5H. This standardized commercial PZT is widely used as a high-precision displacement actuator due to its stable performance, precise control, and cost-effectiveness. The PZT sensor dimensions are 2 × 3 × 10 mm. Consequently, the FBG-FP cascaded fiber-optic sensor is designed with an FP cavity length of 10 mm to achieve maximum detection sensitivity within the constrained structure.

The fabrication of the FBG-FP is carried out first. The selected FBG parameters are as follows: grating length of 10 mm, central wavelength of 1549.954 nm, bandwidth of 0.228 nm, and maximum reflectivity of 92.92%. The FBG is cleaved at its midpoint using a fiber cleaver to obtain two FBG segments of equal length. A 10 mm single-mode fiber is fusion-spliced between the two FBG segments using a fiber fusion splicer to form the FP cavity, completing the fabrication of the FBG-FP. Subsequently, the FP cavity of the FBG-FP sensor is bonded and secured to the PZT sensor using epoxy resin. The fabricated sensor and its spectral diagram are shown in Figure 10. The spectrum was obtained using an AQ6370B optical spectrum analyzer provided by YOKOGAWA (Tokyo, Japan).

### 3.3. Experimental Study of Sensor Detection Characteristics

Prior to current measurement with the fiber-optic current transformer, the sensing probe’s detection bandwidth is tested. A digital signal generator directly supplies a fixed 500 mV AC voltage to the sensing probe. The output frequency is incrementally adjusted from 50 Hz (in 50 Hz steps) to 1 kHz, followed by 0.5 kHz steps until resonance occurs. The frequency is further increased until resonance fully dissipates. The amplitude–frequency characteristic curve of the sensor is shown in Figure 11. Experimental results reveal that the sensor’s output signal peak-to-peak value increases at 7 kHz, with distinct resonance observed at 11 kHz. Thus, the sensor’s detection bandwidth is determined as 0–7 kHz, fully sufficient for power-frequency current detection.

Piezoelectric ceramic materials exhibit hysteresis, which introduces nonlinearity in output displacement and affects sensor measurement performance. However, hysteresis characteristics are strongly dependent on the applied voltage. To investigate the hysteresis of the PZT under low-voltage driving signals, a digital signal generator supplies a DC driving voltage signal ranging from 0 to 1 V with a step size of 0.1 V. The DC component voltage amplitude of the FBG-FP sensor output is recorded at each voltage level. At 0 V, the central operating wavelength of the narrowband optical signal is positioned at the optimal working point Q. Experimental results (Figure 12) show nearly overlapping curves for voltage increase and decrease, indicating negligible hysteresis in the PZT sensor under low-voltage driving signals.

**Figure 13:**
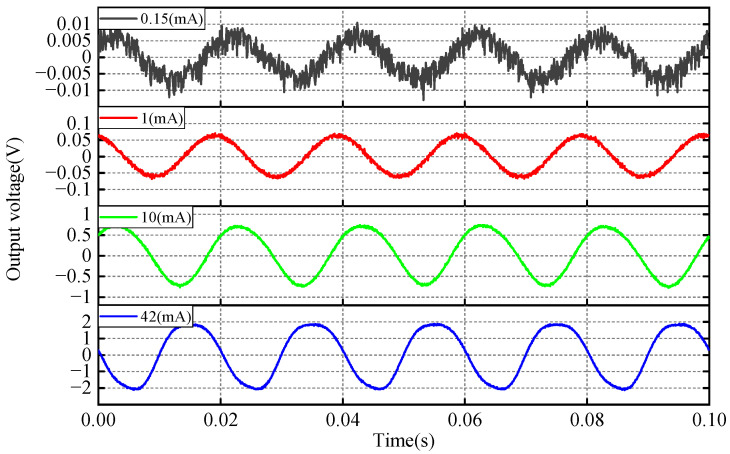
Current test results.

A current measurement system is constructed based on Figure 9, comprising a signal generator, measured busbar, resistors, the FBG-FP sensor, broadband light source, fiber-optic FP tunable filter, photoelectric converter, and oscilloscope. The principle involves inducing a voltage in the magnetic core’s coil when current flows through the busbar. This voltage drives the PZT to generate strain, altering the FP cavity length. The demodulation system reconstructs the current signal and displays it on the oscilloscope. To generate small currents, a 250 Ω resistor is connected in series with the busbar (inherent resistance: 0.88 Ω). The voltage across the resistor is measured by the oscilloscope, and the actual current is calculated by dividing this voltage by the resistance. Output voltage waveforms under different currents are shown in Figure 13. When the measured bus current is 0.15 mA, the sensor’s output signal exhibits a peak-to-peak value of 20 mV, with a system signal noise of 5 mV. For this condition, the signal-to-noise ratio (SNR) is 6.02 dB. When the measured bus current decreases further, the sensor’s output signal peak-to-peak value reduces accordingly, causing the SNR to fall below 6 dB, and the interference of system noise on current detection accuracy increases; thus, 0.15 mA is defined as the minimum measurable current of the FBG-FP sensor. At 1 mA and 10 mA, the output voltage waveforms exhibit good fidelity. When the current reaches 42 mA, the output voltage peak-to-peak value reaches 3.8 V, beyond which waveform distortion becomes significant. This defines the maximum measurable current, resulting in a measurement range of 0.15 mA to 42 mA for the FBG-FP sensor. According to Equation (33), the coil output voltage is only 1.58 V at a busbar current of 1 A. Combined with the low-voltage hysteresis analysis, the sensor can accurately reconstruct the measured current signal within the 0.15 mA–42 mA range.

**Figure 14:**
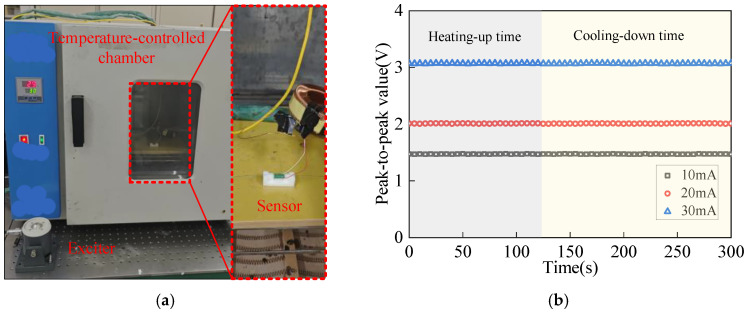
Experimental results of different current values under dynamic temperature conditions: (**a**) experimental system diagram, (**b**) experimental results.

The aforementioned experimental study presents the results of sensor current testing conducted under room-temperature conditions. Subsequently, this paper investigates the sensor’s current detection performance under dynamic heating (20 °C to 50° C) and cooling using a temperature-controlled chamber. As shown in Figure 14a, the system includes the current detection setup within the temperature-controlled chamber. A vibration exciter is installed on the experimental platform and connected to the chamber to simulate external vibration interference in the sensor’s operating environment. The measured bus is introduced through a top aperture in the chamber.

During heating tests, the chamber door is closed to raise the internal temperature. For cooling, the door is opened, and the heat source is turned off. Throughout the experiments, the vibration exciter operates cyclically at frequencies between 0.1 and 2 kHz. Figure 14b illustrates the variation in the peak-to-peak signal amplitude during heating and cooling for different measured currents. The chamber’s internal temperature rises from 20 °C to 50 °C in 2 min and cools to room temperature in 3 min. Experimental results demonstrate no significant variation in the sensor’s output signal amplitude, indicating that the proposed temperature compensation method effectively enables accurate current detection across varying environmental temperatures.

### 3.4. Prospects for FBG-FP Cascaded Fiber-Optic Current Transformers

In power system applications, current transformers operate in distinct scenarios with significantly varying requirements for current measurement ranges. Experimental results indicate that the designed sensor achieves a current measurement range of 0.15 mA to 42 mA under the configuration where the high-permeability magnetic core is fully wound with coils to drive the PZT. According to Equation (33), the number of coil turns exhibits a linear correlation with the output voltage signal amplitude. To address large-scale current detection requirements, the effective measurement range of the FBG-FP cascaded current transformer can be proportionally extended by reducing the number of coil turns on the magnetic core. This proportional adjustment enhances the detection range and engineering applicability of the proposed fiber-optic current transformer, enabling adaptability to broader operational conditions in practical power systems.

## 4. Conclusions

This paper proposes a fiber-optic current transformer based on an electrostrictive material-coupled FBG-FP cascaded structure, aiming to address the limitations of conventional fiber-optic current transformers, such as their low sensitivity, poor temperature and vibration resistance, and the structural complexity of a GMM coupling fiber sensor. A stacked PZT driving structure was adopted, combined with high-permeability magnetic materials, to achieve high-sensitivity detection of micro-current signals. Experimental results demonstrate a detection bandwidth of 0–7 kHz, a current measurement range of 0.15 mA to 42 mA, and a minimum detectable current of 0.15 mA. Furthermore, the integration of FBG-FP quadrature intensity demodulation technology with PID closed-loop control to dynamically adjust the central operating wavelength of the sensor’s driving optical signal effectively compensated for temperature-induced drift, significantly enhancing the sensor’s stability and anti-interference capability. Overall, this study provides a compact, high-precision, and environmentally robust solution for current detection in power systems, exhibiting substantial engineering application value.

## Figures and Tables

**Table 1:** Performance parameters of common PZT piezoelectric ceramics.

Types of Ceramics Include the Following:	PZT-4	PZT-5A	PZT-5H	PZT-8
Mechanical Quality Factor	500	75	65	1000
Piezoelectric Constant d31	−123	−171	−274	−93
Piezoelectric Constant d33	289	374	593	218
Curie Temperature	300	350	190	300
Density	7500	7500	7750	7600

## Data Availability

The original contributions presented in the study are included in the article; further inquiries can be directed to the corresponding author.
